# Genome-scale analyses and characteristics of putative pathogenicity genes of *Stagonosporopsis cucurbitacearum*, a pumpkin gummy stem blight fungus

**DOI:** 10.1038/s41598-020-75235-x

**Published:** 2020-10-22

**Authors:** Qian Zhao, Jianzhong Wu, Liyan Zhang, Chao Yan, Shukun Jiang, Zhugang Li, Dequan Sun, Yongcai Lai, Zhenping Gong

**Affiliations:** 1 Heilongjiang Academy of Agricultural Sciences, Harbin, China; 2 College of Agriculture, Northeast Agriculture University, Harbin, China

**Keywords:** Fungal genomics, Pathogens

## Abstract

Outbreaks of gummy stem blight (GSB), an emerging seed pumpkin disease, have increased in number and have become more widespread in recent years. Previously we reported that *Stagonosporopsis cucurbitacearum* (*Sc.*) is the dominant fungal cause of pumpkin seedling GSB in Northeast China, where it has greatly reduced crop yields in that region. Here, high-throughput whole-genome sequencing and assembly of the *Sc.* genome were conducted toward revealing pathogenic molecular regulatory mechanisms involved in fungal growth and development*.* Zq-1 as representative *Sc.* strain, DNA of Zq-1was prepared for genomic sequencing, we obtained 5.24 Gb of high-quality genomic sequence data via PacBio RS II sequencing. After sequence data was processed to filter out low quality reads, a hierarchical genome-assembly process was employed that generated a genome sequence of 35.28 Mb in size. A total of 9844 genes were predicted, including 237 non-coding RNAs, 1024 genes encoding proteins with signal peptides, 2066 transmembrane proteins and 756 secretory proteins.Transcriptional identification revealed 54 differentially expressed secretory proteins. Concurrently, 605, 130 and 2869 proteins were matched in the proprietary databases Carbohydrate-Active EnZymes database (CAZyme), Transporter Classification Database (TCDB) and Pathogen–Host Interactions database (PHI), respectively. And 96 and 36 DEGs were identified form PHI database and CAZyme database, respectively. In addition, contig00011.93 was an up-regulated DEG involving ATP-binding cassette metabolism in the procession of infection. In order to test relevance of gene predictions to GSB, DEGs with potential pathogenic relevance were revealed through transcriptome data analysis of *Sc.* strains pre- and post-infection of pumpkin. Interestingly, *Sc.* and *Leptosphaeria maculans* (*Lm.*) exhibited relatively similar with genome lengths, numbers of protein-coding genes and other characteristics. This work provides a foundation for future exploration of additional *Sc.* gene functions toward the development of more effective GSB control strategies.

## Introduction

GSB, one of the most serious and common diseases affecting cucurbits production, is caused by fungi of the family *Didymellaceae*, including *Stagonosporopsis cucurbitacearum* (*Sc.*), the focus of this study. GSB mainly infects aboveground parts of cucurbit plants, resulting in damage that generally first appears as necrotic spots on leaves. Rapid spread of *Sc.* to neighboring plants often leads to extensive crop damage, with incidence rates ranging from 15 to 50%^1–4^. Due to past long-term application of chemical fungicides, evolution and gene mutation of GSB-causing fungi have resulted in development of fungicide resistance that is now seriously undermining GSB control efforts. As a result, insidious and rapid *Sc.* spread from an infested field to nearby uninfected areas now leads to increasingly widespread infestations that cannot be controlled, seriously compromising overall yield and quality of cucurbit crops^5^. Therefore, new strategies are urgently needed to combat this threat, with genomics data sought as a powerful information resource toward achieving this goal. However, since no complete *Sc.* genome sequence has yet been reported, most available genetic information with regard to this plant pathogen is limited to data obtained from past studies of spatial genetic structure and population dynamics^6^, genetic structure and evolutionary analysis^7^, diversity analysis^8–10^, rapid diagnosis^2^, host range^11,12^, host resistance genes^13,14^ and development of efficient fungicides^15,16^. While this information has been helpful, a genome-scale level of information would dramatically expand our ability to understand pathogenic *Sc.* mechanisms.

Whole-genome sequencing and genome-wide analysis have already been completed for thousands of fungi^17^ and are particularly important for understanding fungal pathogenic mechanisms. In 2005, the draft genome sequence of *Magnaporthe grisea* was annotated and released, revealing a variety of secreted proteins, including new virus-related genes and enzymes involved in secondary metabolism. Such information has greatly enhanced our understanding of fungal life processes and our ability to predict fungal pathogenic mechanisms^18^. As a separate project involving the sequencing and annotation of the genome of a strain of *Fusarium graminearum*, a major pathogen of cultivated grain, Cuomo (2007) discovered a highly polymorphic region containing a cluster of genes related to plant-fungus interactions^19^. Subsequently, Kronstad (2008) reviewed partial genome sequence results for *Ustilago maydis* and demonstrated the power of comparative genomics to determine genomic similarities and differences between species as a means of harnessing genomic information from diverse fungi to enhance understanding of fungal processes^20^. More recently (2017), systematic bioinformatics analysis of *bZIP* genes of rice smut pathogenic fungus (*Ustilaginoidea virens*) for detection of bZIP regions in predicted genes discovered 11 *UvbZIP* genes associated with fungal infection as an example of how bioinformatics tools can reveal genetic structures, motifs and phylogenetic relationships^21^. In 2018, six keratinase coding genes were identified within the *Rhizoctonia cerealis* genome (designated *RcCUT1–RcCUT6*). Subsequent analysis of fungal gene expression patterns during infection led to the discovery of *RcCUT1,* a toxic factor produced by fungi^22^. Taken together, these studies illustrate the power of whole-genome sequencing and genome-wide analysis to greatly facilitate the discovery of fungal pathogenic molecular regulatory networks and disease-causing genes. Indeed, such methodologies have already greatly expanded our understanding of fungal pathogenic processes, prompting us to use these techniques to study *S. cucurbitacearum* in this work.

In the present study, high-throughput sequencing technology was used for the first time to sequence the genome of *S. cucurbitacearum* isolate zq-1, an *Sc.* isolate obtained from a cold region of China. A high quality whole-genome fine map was obtained after genome assembly with a total size of 35.28 Mb. Based on the complete *Sc.* genome sequence, differentially expressed *Sc.* genes at the transcriptional level before and after *Sc.* infection of pumpkin were analyzed, resulting in identification of genes of numerous secreted proteins, plant cell wall degradation enzymes and secondary metabolic enzymes that may play important roles in GSB pathology. Ultimately, the *Sc.* genome sequence will likely provide additional insights for development of new strategies to control GSB and other fungal diseases of crops.

## Results

### Strain identification

By morphological characteristics combined with rDNA internal transcriptional interval (ITS) sequence analysis, the strain of Sc. was identified. As observed for other pathogenic fungi of pumpkin, infection of pumpkin leaf with *Sc.* can produce disease spots (Supplementary Fig. S1A, quote from https://extension.umn.edu/diseases/gummy-stem-blight-and-black-rot)*.* Under suitable conditions, *Sc.* mycelia growing on PDA medium (Supplementary Fig. S1B) or on pumpkin leaves produced similar infection nails (Supplementary Fig. S1D), which germinated from fungal bud tubes by 36 h post-infection (Supplementary Fig. S1C).

### Characteristic of genome

**Figure 1:**
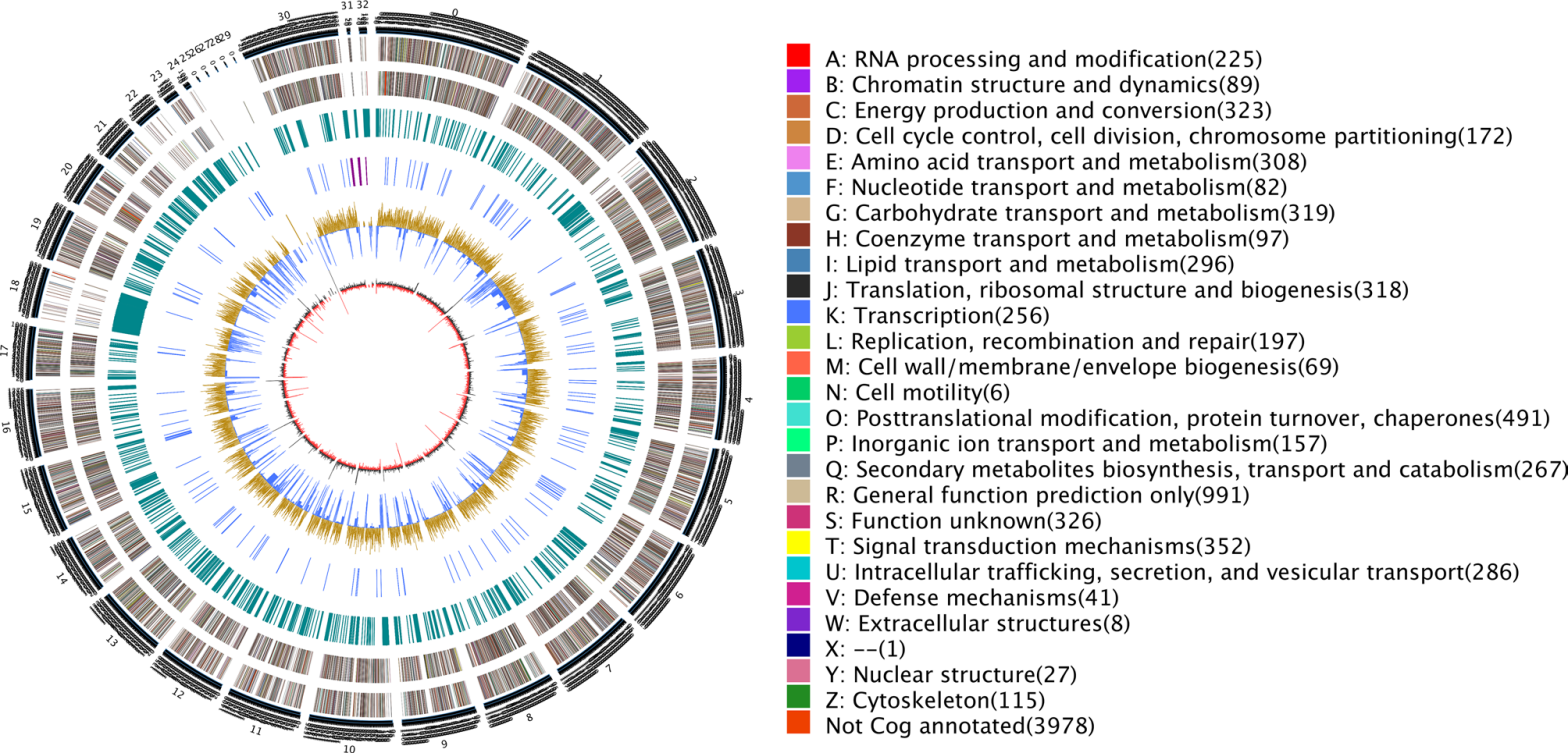
Genomic map of *Stagonosporopsis cucurbitacearum *(*Sc*.). The outermost circle is a marker of genome size with each demarcation representing 5 kb that shows the entire genome size of *Sc.* zq-1 of 35.28 Mb. The second and third circles from the outside show positions of genes coded by plus and minus strands of the genome, respectively. The function and gene number annotated by COG are listed on the right of the figure whereby each function corresponds to a color and each color corresponds with a different COG functional classification. The fourth circle shows repeating sequences. The fifth circle shows genes coding for tRNA and rRNA, where blue and purple represent tRNA and rRNA, respectively. The sixth circle shows the GC content, the light yellow part shows GC content is higher than the mean GC content of genome, the higher the peak value is, the bigger the difference is, and the blue part shows GC content is lower than the mean GC content of genome; The innermost circle shows the GC-skew, with dark gray representing regions where G is greater than C and red representing regions where C is greater than G. Circos (https://circos.ca/) was used for drawing program.

The genome of *Sc.* was assembled from data generated using the Single Molecule Real-Time (SMRT) sequencing strategy, an effective method introduced by Pacific Biosciences to decode important sequence regions that can facilitate the generation of gapless eukaryotic genome sequences^23^. After obtaining 527,073 subreads, a total of 5.24 Gb of genome sequence was obtained. High quality of the 10-kb library was confirmed using subreads distribution analysis (Supplementary Fig. S2) that provided a subreads N50 number of 12,559 sequences and a mean sequence length of 9946 bp (Supplementary Table S1). Subreads were assembled into 33 scaffolds (N50, 1.57 Mb) to generate a total genome length of 35.28 Mb, a genome size smaller than that of *Lm.* (45.12 Mb; genome assembly # ASM23037v1) (Table 1). We have performed BUSCO to evaluate genome completeness from the annotation (Supplementary Fig. S3).A Circos-plot, used to display genome assembly information (Fig. 1), shows thirty-three scaffolds arranged in the outermost circle and displays the predicted 9844 protein-coding genes, for a gene density of 279 genes per 1 Mb that represents a total genome coverage rate of approximately 48.22%. Sequence analysis predicted 176 secreted proteins and 14 pseudogenes from the genome sequence (Table 1). BLAST comparisons were conducted between *Sc.* gene sequences and multiple functional databases to obtain functional annotation results for 9562 genes (Supplementary Table S2). It is noteworthy that 8913 putative protein-coding genes were detected within the RNA-seq data.

**Table 1:** The basic characteristics of *Sc.* and *Lm.*

Features	*Leptosphaeria maculans*	*Stagonosporopsis cucurbitacearum*
Assembly size (Mb)	45.12	35.28
Scaffolds	76	33
GC (%)	44.10	50.20
Repeated sequences (%)	34.20	9.60
Protein-coding genes	12,469	9844
Gene density (genes per Mb)	276	279
Mean gene length (bp)	1323	1728
Secreted proteins	–	759
tRNA	–	176
Pseudogenes	–	14
No. of superContigs (sCs)	76	33
SC N50 (Mb)	1.80	1.57
No. of predicted genes	12,469	9844
Average gene length (bp)	1323	1728

### Repetitive DNA sequence and DNA methylation

**Figure 2:**
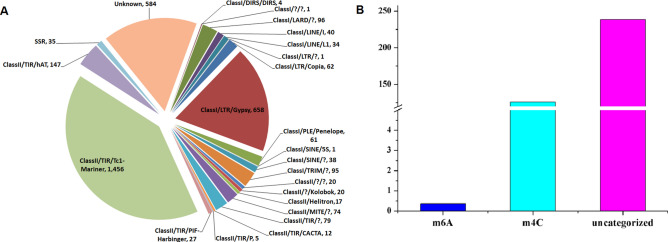
Statistical analysis of repeat sequences and DNA methylation in *Sc.* genome. (**A**) The percentage of different types of repeat sequences in the *Sc.* genome. (**B**) Statistical analysis of the methylation sites of candidate DNA in the main sequence of *Sc.* genome, where m4C and m6A represent 4-methyl cytosine and 6-methyl adenosine, respectively.

**Figure 3:**
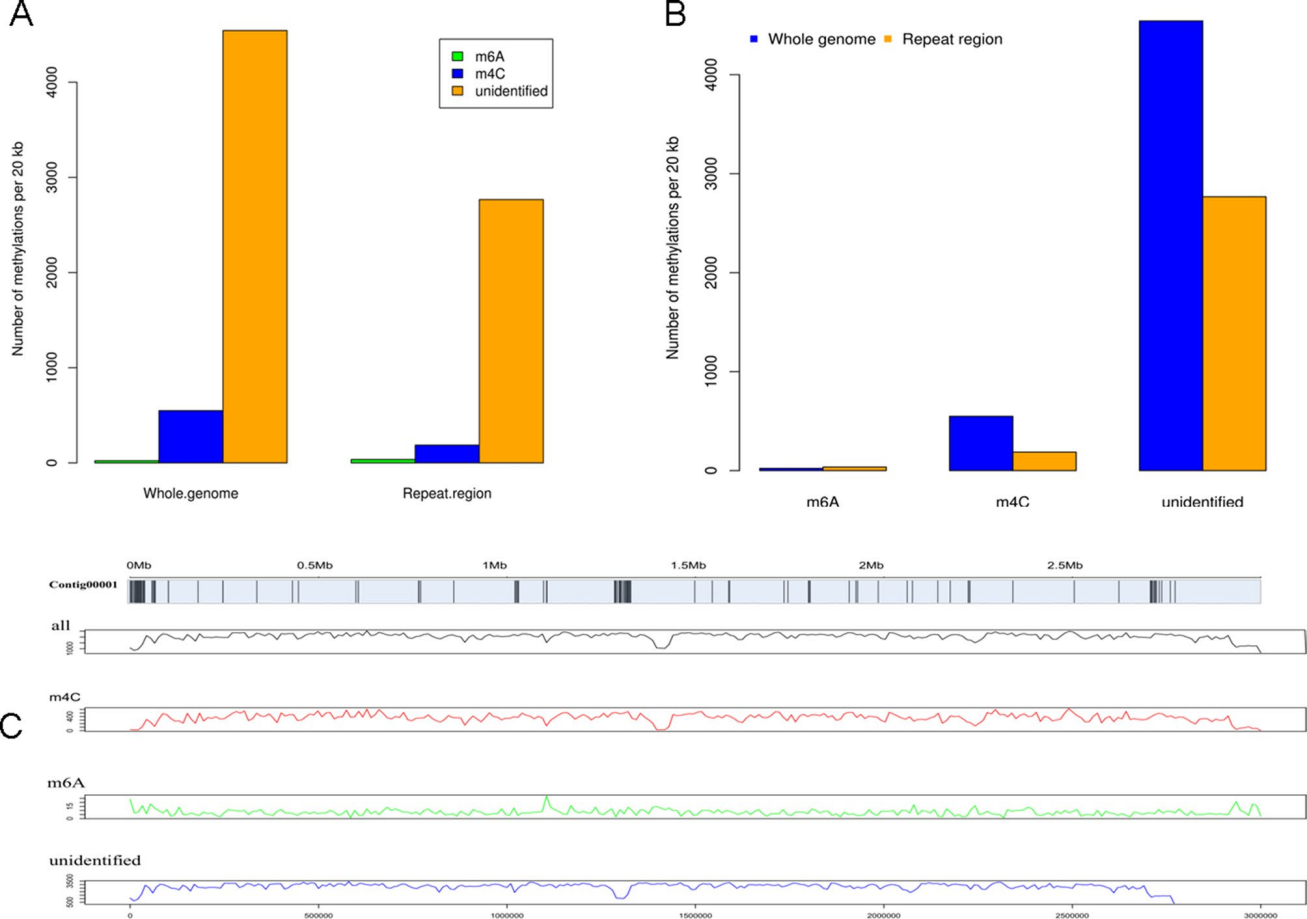
DNA methylation analysis of *Sc.* genomic. (**A**) The distribution of repeat sequences in the scaffold and different DNA methylation types. The histogram represents the distribution of repeating elements. All data were analyzed to produce the statistical result indicated within the 20-kb window. (**B**) Calculation of the number of different types of methylation per 20 kb across the entire *Sc.* genome. (**C**) Distribution of repeating elements.

Repetitive DNA sequences play important roles in genome structure, gene evolution and fungal functions^24^. Here, we identified 3,387,350 bp of repeat sequences in the *Sc.* genome that accounted for 9.6% of the genome, including LTR retrotransposons, DNA transposons, tandem repeat sequences and other unclassified transposons (Fig. 2A). DNA methylation plays an important role in biological processes, such as in genomic imprinting and regulation of gene transcription^25^. In recent years, more and more reports on DNA methylation have been reported for fungi, while numerous reports exist describing this phenomenon in higher plants and animals^26^. In order to detect 4-methyl-cytosine (m4C) and 6-methyl-adenosine (m6A) methylation events within the *Sc.* genome, SMRT technology was used. Subsequently, 1,228,069 m4C and 30,883 m6A sites in the *Sc.* genome were identified (Fig. 2B), with most methylation sites (23,845,215) uncategorized. Ultimately, 99.71% of categorized DNA methylation events were categorized as m4C, whereas m6A only accounted for 0.29% of events. However, compared with m6A, m4C, DNA methylations occur with low frequency in the regions of repetitive elements (Fig. 3A–C).

### Comparative genome

The evolutionary relationship between *Sc.* and analogous species was studied using BLAST searches against the Nr database (Supplementary Fig. S4). As shown in Supplementary Fig. S4, *Sc.* had higher genomic similarity with *Phaeosphaeria nodorum* (18.56%) and *Leptosphaeria maculans* (18.39%), while only *Leptosphaeria maculans* (*Lm.*) had genome datas (FA format) and genome annotation files (GFF format) in the public database. Considering the results of the analysis of the public database information reported so far, although the similarity of only 18% between *Sc.* and *Lm.*, the similar sequences still provide data support for genome annotation and further functional gene research of *Sc.* In terms of their relatedness based on homology (similarities of nucleotide composition and gene sequences) and their evolutionary relatedness (based on distances between genes and gene positioning patterns of biological systems), the results of analysis of both types of comparisons between the two plant pathogenic fungi are shown in Table 1.

Although genome similarity between *Sc.* and *Lm.* was relatively high, some differences were observed: the genome of *Sc.* was smaller (35.28 Mb) than that of *Lm.* (45.12 Mb) and had lower GC content (44.1%) than did that of *Lm.* (50.2%); The numbers of genes encoding proteins of *Sc.* and *Lm.* were 9,844 and 12,469, respectively; the respective average gene lengths of *Sc.* and *Lm.* were 1728 bp and 1323 bp, with N50 values of 1.80 Mb and 1.57 Mb, respectively. Notably, the most obvious difference between *Sc.* and *Lm.* was due to the larger number of scaffolds and greater proportion of repeated sequences for *Lm.* which were 2.3 times and 3.56 times higher than respective *Sc*. values.

**Figure 4:**
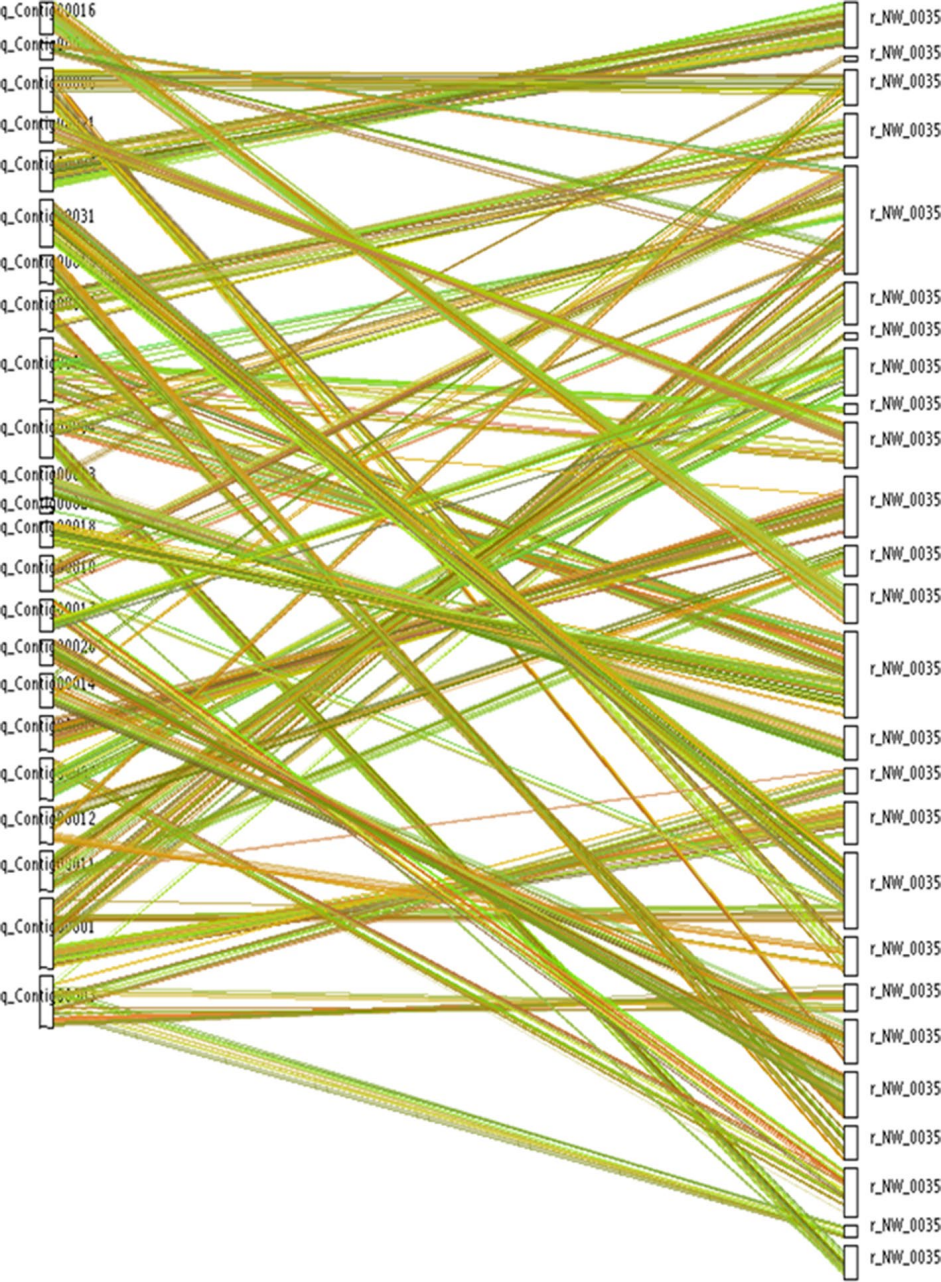
Comparative genome collinearity analysis of *Sc.* and *Lm.* The assembled genome is shown on the left and the reference genome on the right. Analogous genes between species are joined with a line. Thus, the homology of coding sequence and structure between modular genomes is established, whereby mapping information of known genomes is used to locate genes within other genomes that, in turn, reveals potential functions of genes, evolutionary relationships between species and internal structures of genomes. MCScanX (https://chibba.pgml.uga.edu/mcscan2/MCScanX.zip) was used for drawing program.

BLAST comparisons of protein sequences of *Sc.* and *Lm.* genomes revealed a collinear relationship at the nucleic acid level that reflected differences between species in relative positions of analogous genes within their genomes (Fig. 4). In general, comparative genetic studies show that genes and sequences of many fungal genomes are highly conserved at the genetic map level, but the level of collinearity is less conserved at the micro mapping level (with the exception of very closely related species that maintain good collinearity). In addition, MCScanX-based analysis was also used to conduct collinearity analysis of amino acid sequences of both genomes and demonstrated a similar linear relationship between *Sc.* and *Lm*. genes. Here, the collinearity analysis was performed on the genome, for strains *Lm.* and *Sc.* homologous proteins, and the collinearity fragments were obtained with the help of the relative position of genes in the chromosome. All these fragments provide support for the detection of genome-wide gaps in the coding genes for unknown functional protein, which will be one of the continuing research focuses of this research.

### Differentially expressed gene analysis

After sequencing and quality control, a total of 44.62 Gb clean data were obtained, with clean data per library approaching 7.15 Gb, and the Q30 base percentage were greater than 92.38% (Supplementary Table S3). During the detection of differentially expressed genes (DEGs), Fold Change ≥ 2 and FDR < 0.01 were used as the screening criteria (Table 2), 319 DEGs between *Sc.1* (the average value of three replications of T01, T02 and T03) and *Sc.2* (the average value of three replications of T04, T05 and T06) were obtained, in which 190 and 129 genes were down- and up-regulated (Supplementary Fig. S5). 58 DEGs were identified as pathogenicity genes involved in multiple molecular functions within biological processes, such as hydrolytic enzyme, protein kinase, phosphatase, cellulase, etc. Among them, 22 DEGs were associated with transmembrane transport (GO:0055085, GO:0003333 and GO:0005215) and 16 DEGs were associated with hydrolytic enzyme (GO:0016787, GO:0004553 and GO:0016810). In addition, 20 genes may be directly or indirectly involved in growth and pathogenicity of *Sc.* (Supplementary Table S4), since they belong to biological processes of cellulase activity (GO:0008810), phosphorelay sensor kinase activity (GO:0000155), proteolysis (GO:0006508) and interaction with host via protein secreted by type II secretion system (GO:0052051). Hierarchical cluster analysis of the abovementioned 58 DEGs based on log10 (RPKM) values showed that the overall gene expression pattern could be divided into several clusters based on DEGs expression levels for T01, T02, T04, and T05; this result demonstrates obvious differences in DEGs between *Sc.*1 and *Sc.*2, a result consistent with Pearson correlation coefficient r results and comparison diagram analysis of the FPKM density distribution (Supplementary Fig. S6). The most significant KEGG pathways associated with DEGs are shown in Supplementary Fig. S7; DEGs between *Sc.1* and *Sc.*2 were assigned to 7 KEGG pathways.

**Table 2:** Differentially expressed gene results of *Sc.* 1 (before infection) and *Sc.* 2 (after infection).

T1 count	T2 count	T3 count	T4 count	T5 count	T6 count	FDR	log2FC	Regulated
20	17	29	993	1304	181	0.000448	4.848797	Up
79	263	449	0	0	0	0.00024	− Inf	Down
2693	5952	5843	193	161	607	0.000164	− 3.76636	Down
201	93	91	1878	2385	861	0.001049	3.510969	Up

*Count* the Count value of gene expression of the samples, *FDR* error detection rate, *Log2FC* logarithm value of expression amount difference multiples, *Regulated* up- or down-regulated gene.

### Secreted proteins and effector factors

**Figure 5:**
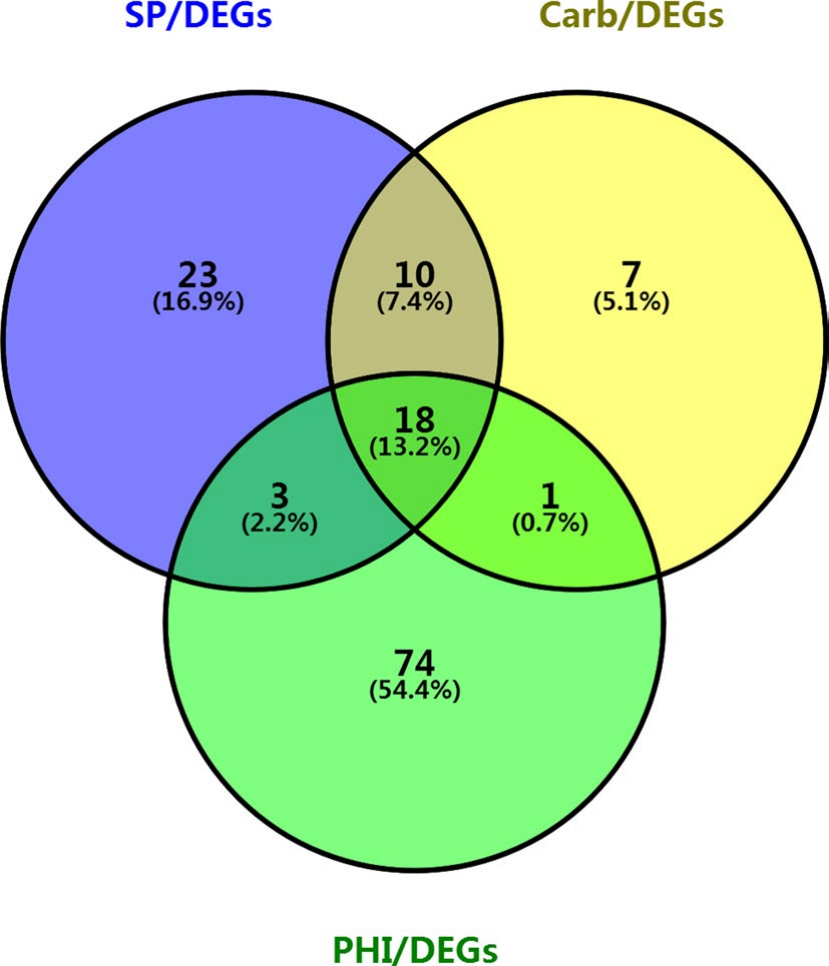
SP/DEGs, Carb/DEGs and PHI//DEGs Wayne figure. SP is abbreviation of secreted protein, DEGS is abbreviation of differentially expressed genes, Carb is abbreviation of CAZymes , PHI is abbreviation of pathogen–host interaction gene. TBtools was used for drawing program (https://github.com/CJ-Chen/TBtools/releases).

Annotation of the *Sc.* genome yielded a total of 7238 proteins based on Pfam database matches. Domain calling analysis revealed that more than 2738 domain types exist in the *Sc.* proteome, which include effectors secreted by pathogens that may play critical roles in plant suppression of pathogen proliferation^27^. Among these effectors, 1024 signaling peptide proteins were identified. After removal of transmembrane helix proteins from this list, 759 secreted proteins of the *Sc.* Genome were identified. Of these proteins, secreted protein functional domains with the highest frequency of representation were counted to reveal that the largest number of structural domains belonged to glycosyl hydrolase family 61 (PF03443.9), followed by zinc carboxypeptidase (PF00246.19), the WSC domain (PF01822.14), the subtilase family (PF00082.17) and the kelch motif (PF13854.1). Functional domains with lowest frequency of representation included chitin recognition protein (PF00187.14) and fungal cellulose-binding domain (PF00734.13) (Supplementary Table S5). Ultimately, 54 predicted secretory proteins were subsequently shown to be differentially expressed (Fig. 5 and Supplementary Table S6).

### Analysis of PHI related genes and cell degrading enzymes

PHI is an important database repository of pathogen-host interaction factors and contains a large number of important pathogenicity genes that are useful for analyzing the pathogenic potential of pathogen virulence factors^28^. After comparing the entire genome of *Sc.* to the PHI database, 2869 genes with high homology were detected, accounting for 29.14% of the total number of genes in the *Sc.* genome, of which 96 genes were differentially expressed (Fig. 5, Supplementary Table S7).

Notably, four classes of DEG annotations were identified in PHI that included secretome, putative carbohydrate-active enzymes (CAZymes) and secondary metabolic processes, all of which indicate that successful phytopathogenic fungi can break down and utilize plant cell wall polysaccharides by secreting CAZymes. Such plant cell wall-degrading enzymes are especially relevant to fungal pathogenicity, due to their important roles in penetration and successful infection of plant hosts. In this study, 36 DEGs of the total 605 genes encoding carbohydrate enzymes were predicted to have pathogenic relevance based on matches with CAZyme database proteins (Fig. 5 and Supplementary Table S8).

In the *Sc*. genome, approximately 37.69% of potential secreted proteins (278/605) were predicted to be CAZymes, demonstrating that *Sc.* may employ a large group of CAZymes to digest host cell walls during invasion. Interestingly, one family of CAZymes, the glycoside hydrolase GH109 family, was highly enriched (Supplementary Table S9); one member of this family is known to biochemically function as an α-*N*-acetylgalactosaminidase (α-NAGAL), which can cleave the bond linking terminal alpha-linked *N*-acetylgalactosamine group to the A blood group antigen protein^29^. Notably, we found that *Sc.* encodes 10 putative GH109 genes, a higher number than observed for most fungi, including *Magnaporthe oryzae, Botrytis cinerea* and *Neurospora* crassa^30^. Thus, these data suggest that expansion of the GH109 family in *Sc.* may have endowed the pathogen with the ability to overcome pumpkin lectin-mediated resistance to infection.

The GH18 family, the third largest group within the glycoside hydrolyase (GH) family, contains many fungal chitosan enzymes that function along with other cell wall-degrading enzymes to ultimately transform and recycle the fungal cell wall^31^. Ten potential GH18 family coding genes from the *Sc.* genome were identified and appear to code for secreted proteins, due to their homology with pathogenicity or virulence genes PHI 144 and PHI 1178 (reduced virulence) within the PHI database (Supplementary Table S10). As carbohydrate enzymes have been found among many important enzyme families, analysis of protein families may provide insights regarding the regulation of expression of carbohydrate enzyme-coding genes of pathogenic fungi.

Of annotated carbohydrate esterase genes described in this work, we found a total of 74 genes belonging to two major subfamilies, CE1 and CE10. Both families encode proteins exhibit activities characteristic of carboxylesterases and endo-1,4-β-xylanases. Meanwhile, although 47 potential carbohydrate-binding module (CBM) proteins that digest complex carbohydrates extracellularly^32^ have been identified in fungal genomes, unexpectedly only one CBM protein, CBM21, was found in the *Sc.* genome. Nevertheless, this observation may be relevant to pathogenicity, since many phytopathogenic fungi generate abundant quantities of CBM21^32^.

### Secondary metabolic enzyme

During interactions between fungi and host plants, many secondary metabolites, such as fungal toxins, have been shown to participate in and enhance the virulence of pathogenic fungi. Biosynthesis of fungal secondary metabolites mainly relies on five key enzymes: polyketone synthase (PKS), non-ribosomal peptide synthase (NRPS), non-ribosomal peptide synthase-polyketide synthase hybrid (hybrid NRPS-PKS), dimethyl tryptophan pyrophosphate synthetase (DMATS) and terpene cyclase (TC). Here a total of 3327 *Sc.* genes related to functional pathways within metabolic process (GO: 0008152) were found. Of these genes, 86 metabolically relevant genes were found to be differentially expressed (40 up-regulated and 46 down-regulated genes) using transcriptome data. Of the 40 up-regulated genes, Contig00004.509 showed the highest level of expression, which was annotated with hydrolase activity in GO:0016787, and cellulose 1,4-beta-cellobiosidase activity in GO: 0016162, followed by Contig00031.5 (Catalytic activity, GO:0003824) and Contig00015.96 (oxidoreductase activity, GO: 0016491).

**Figure 6:**
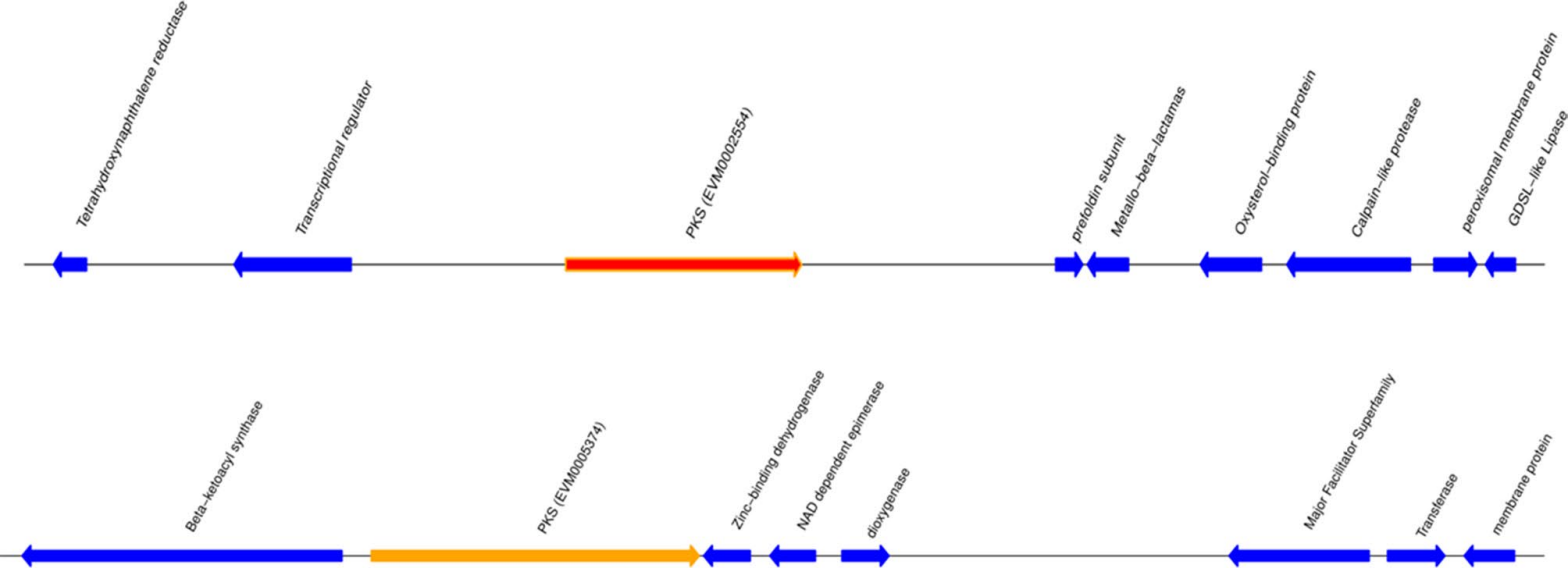
Pigment gene cluster. Red represents pigment genes, blue represents other genes in the cluster and the forward arrow points to the right.

The *Sc.* genome encodes 34 non-ribosomal peptide synthetases (NRPSs), 11 type III polyketide synthases (T3PKSs), 44 type I polyketide synthases (T1PKSs), 85 NRPS-like enzymes, 29 terpene synthases and 3 indole synthases, but lacks PKS-NRPS hybrid proteins and PKS-like proteins (Supplementary Tables S11, S12). This finding is relevant to pathogenicity, since pigments are an important group of secondary metabolites that protect pathogens from host oxidative stress damage during invasion. Notably, the *Sc.* genome encodes multiple putative PKSs that likely participate in pigment production (Fig. 6 and Supplementary Table S12).

### Transporters

Amino acid sequence BLAST comparisons conducted between *Sc.* sequences and the TCDB database predicted a total of 130 transport-related genes, of which the largest number (45 genes) were related to transporters (one-way transporter, cotransporter, reverse transporter) and 44 genes were related to P–P-bond-hydrolysis-driven transmembrane transporters (Supplementary Fig. S8). These genes together constitute the main transporter system of *Sc.*, of which eight genes exhibit differential expression before versus after infection and were designated as contig00003.94, contig00009.78, contig00009.120, contig00009.376, contig00013.104, contig00013.149, contig00015.279 and contig00016.184.

A total of 62 genes annotated in PHI database were found to be associated with a total of 44 PHI-base accessions (Supplementary Table S13) and 3 ABC transporter gene proteins (contig00001.338, contig00004.179 and contig00011.394). ABC transporters play an important role in the processing of antifungal toxins and in cytotoxin transport, which are processes that are needed to overcome the toxic effects of anti-cytotoxins produced by the host during fungal invasion^33^. Notably, transcriptome analysis showed that contig00011.93 contained an up-regulated DEG that was involved in ATP-binding cassette metabolism in infected leaves. Thus, transporter genes may be important pathogenic factors involved in *Sc.*-plant interactions*.*

### Expression of predicted pathogenic genes

**Figure 7:**
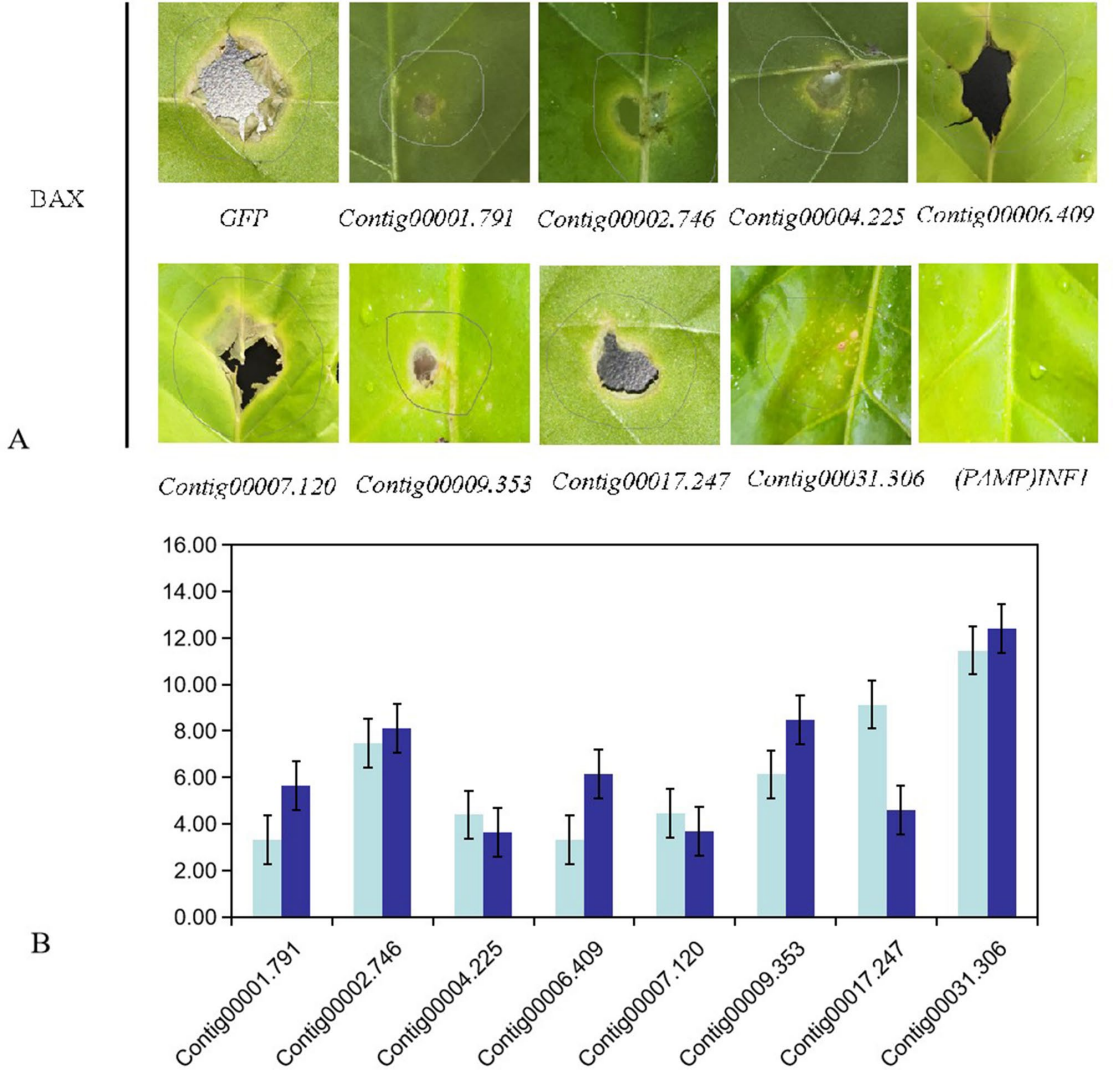
Functional analysis of putative *Sc.* effectors. *Sc.* effectors suppress BAX-induced programmed cell death in *Nicotiana tabacum*. *Nicotiana tabacum* leaves were infiltrated with agrobacterium carrying *Sc.* effector genes. *Phytophthora* oocytes (infestin-1 or INF1) served as positive controls and GFP as negative control. Pumpkin leaves were inoculated with *Sc*. Samples were collected at 0 day and 5 days. Quantitative RT-PCR (qRT-PCR) was used to determine gene expression levels. Values represent the mean ± SD (n = 3 biological replicates). The blue and red bars in figure B represent the gene expression at day 0 and day 5 after inoculation, respectively.

Eight genes were randomly selected from the total 136 predicted pathogenic genes for assessment of gene expression levels (Supplementary Tables S6, S7, S8). Primers used in these experiments are listed in Supplementary Table S14. In order to study the functions of predicted genes, we transiently expressed the eight genes in *Nicotiana tabacum* then screened plants to detect potential effectors that suppressed BAX-triggered PCD (BT-PCD). Our results showed that the eight genes suppressed BT-PCD. As for the cracks after Contig00006.409 and Contig00007.120 produced pathogenicity, this uncontrollable factor may have a slight influence on the results, but it will not affect the overall evaluation (Fig. 7A). This result aligns with qRT-PCR results showing that expression levels of these seven genes increased and one decreased after 5 days of infection of pumpkin leaves (Fig. 7B). Therefore, the results obtained in the field support our predicted results.

## Discussion

### Whole genome sequencing and analysis

Genomics belongs to the scientific branch of applied bioinformatics and requires high quality nucleotide sequences for successful results. Currently, fungal DNA isolation methods mainly include mycelial and spore DNA extraction methods^34^. Due to the large amount of GSB fungal mycelial material obtained from PDA culture, mycelial enrichment was carried out by scraping material directly from the surface of the solid PDA medium followed by grinding of the liquid nitrogen-frozen fungal material and associated agar into a powder. The powder was then subjected to DNA extraction, resulting in isolation of DNA of high purity and integrity for this study.

Genome-wide analysis of plant pathogenic fungi is helpful for understanding evolutionary relationships between pathogens, for detecting disease-related genes and for determining mechanisms that underlie observed variations in virulence, all of which are relevant to controlling fungal diseases of plants^35^. Here, comparisons of sequences within the *Sc.* genome to known genes within multiple databases resulted in prediction of 9844 protein-coding genes, including 2869 pathogen-host interaction-related genes, 605 carbohydrate synthase-coding genes and 130 transporter-coding genes. By studying variations of such genes and how they correspond to variable levels of pathogenicity, clues to interactions between pathogens and hosts may be found. For example, if the virulence of GSB is low only toward a single specific variety of seed pumpkin (but not to other varieties), identification of gene variations in that pumpkin variety could reveal an unknown but important participating host factor that could serve as a gene target for use in development of GSB-resistant seed pumpkin varieties.

In another example, proteins secreted by pathogens often must interact with key plant effectors in order to cause pathogenic effects in plants; therefore, variations in plant effector genes may sometimes undermine plant resistance to pathogens^36,37^. Indeed, most transporter protein families that participate in pathogen-host interactions are likely clues to understanding pathogenic mechanisms. However, based on this dialectical view, the use of bioinformatics tools to deduce functions of predicted genes may also have limitations. For example, although pathogenic genes for *Sc.* interaction with host were identified and classified based on results of BLASTp searches of the PHI database, the expression of several analogous genes varied among different pathogenic organisms; thus these genes require further verification to confirm disease specificity or selectivity. In addition, many identified *Sc.* gene-encoded proteins, such as protein kinases, ABC transporters and major facilitator superfamily (MFS) transporters, are frequently directly or indirectly involved in these processes. Therefore, although some pathogenically relevant protein gene functions have been identified using bioinformatics methods, others genes require further functional verification to confirm their functions.

DNA methylation is an important field of epigenetics in eukaryotes and prokaryotes. Of the three most studied DNA methylation types observed in fungi, m5C, m4C and m6A^38^, only two types of DNA methylation (30,833 sites for 6-mA and 1,228,069 sites for 4-mC DNA methylation) were predicted in the *Sc.* genome using SMRT software. Generally, m6A is considered to be an epigenetic signal involved in DNA–protein interactions, while m4C is considered to be a modification present at lower frequency within regions of repeating elements. In this study, 100 times more m4C sites were observed than m6A sites, indicating that m4C may be generated during transposition of *Sc.* transposons. Nevertheless, SMRT can directly detect DNA methylation through information provided by polymerase kinetics, analysis of GSB DNA methylation confirms that SMRT is a powerful tool for studying epigenetic modifications of fungal genomes.

At present, the absence of genomic studies for *Sc.* has hindered development of reference materials for the study of GSB in real time. However, high-throughput sequencing platforms have made it possible to obtain and analyze whole-genome sequences, especially since third-generation sequencing technologies that are now available are very powerful tools for genomics research and sequence analysis^39^. In this work, the whole-genome sequence was obtained for a cold-adapted strain of *Sc.* using second- and third-generation high-throughput sequencing methods. After data quality control, genome assembly, genome component analysis and DNA methylation analysis were completed, functional annotation ultimately identified numerous possible pathogenic genes that code for cell wall degradation enzymes, protein transport system proteins and pathogen-host interactions. These *Sc.* genome sequence results will facilitate future research efforts to understand GSB pathogenic mechanisms.

### Comparative genomics analysis

In this study, BLAST alignments of genome sequences of various related species resulted in identification of *Lm.* as the closest relative of *Sc.*, with genomic collinearity between the two species aiding discovery of pathogenic and functional genes. Indeed, many fungal genomes contain highly conserved genes and gene sequences that exhibit good collinearity among closely related species (that are separated by small evolutionary distances). For example, sequence homology analysis of genomes of *Cercospora sojina* Hara and other species reported by Luo et al*.* in 2018 demonstrated a close relationship between *C. sojina* Hara and *Dothideomycetes* spp. However, these results conflicted with collinearity analysis results, which demonstrated that *C. sojina* Hara shares greatest collinearity with *Cercospora zeae* Maydis*.* Specifically, scaffold 1 in the genome of strain *Cercospora sojina* Hara is very similar to scaffold 4 in the genome of *Cercospora zeae* Maydis, sharing about two-thirds of similar genetic sequence on one end of the scaffold and one-third of similar sequence on the other end of the scaffold, but on the opposite strand^30^. These differences may explain why the two strains, although they are closely related, are different and illustrate why it is important to conduct genetic similarity analysis of scaffolds.

At the start of this study, none of the genomes of *S. cucurbitacearum*, *Stagonosporopsis citrulli* or *Stagonosporopsis caricae*, all of which cause GSB, had been released. However, due to the fact that the genome sequence for *Lm.* was available and that *Lm.* is highly similar to *Sc.*, the *Lm.* genome was selected for comparative genome-wide analysis against the *Sc.* genome^40^. After the analysis, genomes of *Sc.* and *Lm.* were found to be relatively similar in length (35.28 Mb vs. 45.12 Mb, respectively) with similar numbers of protein-coding genes (9844 vs. 12,469), fueling speculation that they are closely related and probably share common past evolutionary rearrangement events^41^. Moreover, subsequent MCScanX-based analysis revealed a highly conserved collinearity relationship between *Sc.* and *Lm.* Thus, the large number of conserved sequences and highly conserved genome structures between these organisms suggest that the *Lm.* genome sequence will be highly useful for completing gap repair of the *Sc.* genome now that numerous *Sc.* genes have been mapped and structures and functions of numerous genes have been characterized^42,43^. Furthermore, additional results have confirmed relatively high genomic correlation between *Sc.* and *Lm.*, further supporting a high degree of relatedness based on both sequence homology and evolutionary history^44^. Here, comparative genomics was conducted to comprehensively analyze the relationship between *Sc.* and other related species from a whole-genome perspective, groupings of genes with similar functions and characteristics were generated based on homologies to known genes, thus demonstrating that gene functions can be predicted. In addition, these results provide valuable insights for the development of methods to achieve early and rapid diagnosis of GSB and for development of strategies to combat GSB.

### Pathogenic gene analysis

About 70–80% of plant diseases are caused by numerous species of pathogenic fungi that are highly distinct with respect to population differentiation, infection mode, infection structure, pathogenic genes and nutritional strategies^45^. Meanwhile, plant fungal diseases are based on interactions involving host plants, pathogenic fungi and environmental factors. More specifically, pathogenic fungi invade the host by adhering to host surfaces to form infection structures that then cause disease via internal colonization and expansion of the host^46^. Due to the fact that regulation of pathogenic genes occurs throughout the entire infection process, in-depth exploration of plant pathogenic fungal genes and their functions should enhance understanding of pathogenic fungal mechanisms from a molecular perspective. In this way, accurate prevention and control of pathogenic fungi may be achieved through development of target drugs that induce disease resistance in the future.

The plant cell wall is the main physical barrier between plant and pathogen during the infection by plant pathogenic fungi, which invade host tissues by producing a series of cell wall-degrading enzymes (CWDEs)^47^. Here, CAZyme was used as a tool to predict approximately 278 putative secreted proteins in the *Sc*. genome that notably include cell wall-degrading enzymes with relevance to GSB. In the future, prediction of proteins involved in *Sc.* infection structures involved in fungal invasion of pumpkins will also be conducted.

Using RNA-seq technology, scientists have discovered that many secreted proteins, secondary metabolic enzymes and receptor protein-coding genes play important roles in host–pathogen interactions. Here, we found that the disease-causing genes predicted from the special function database were not corresponding with the transcriptional analysis, this was attributed to the fact that transcriptional expression analysis was only the differential expression of genes before and after the host infection, while the expression of pathogenic genes predicted by the database is not significantly different during the treatment period. In addition, the Venn diagram was drew from the three databases and eight infection-related genes that were not significantly differentially expressed at transcriptome level during this treatment period.

In this study, 22 DEGs were detected using GO analysis that was related to transmembrane transport. Although these genes appear to be related to *Sc.* toxin secretion and drug resistance, their specific functions require further verification. One group of transmembrane transport proteins that contains the largest number of efflux pumps, the ABC family of transporters, are primary active transport systems that play an important role in antifungal toxin and cytotoxin transport. During plant host invasion, pathogenic fungi rely on these transporters to counter toxic effects of host-generated anti-cytotoxins^48^. Interestingly, one DEG in this group found here (Contig 00011.93), was an ATP-binding cassette transporter (a type of ABC transporter) that was up-regulated in infected leaves. In addition to transmembrane transport proteins, hydrolytic enzymes of pathogenic fungi were also detected in *Sc.* This is an interesting result, since hydrolytic proteins have important pathogenic roles. Such roles have been demonstrated in studies showing that fungal pathogenicity can be reduced by inhibiting expression of a hydrolytic enzyme gene^48^, possibly because hydrolytic enzymes can participate in the release of fungal defense substances that facilitate pathogen host invasion^49^. In this study, 16 *Sc.* genes were found to be related to hydrolytic enzymes, which may participate in pathogen infection and colonization of host tissues.

As an important means used by pathogenic fungi for absorption of foreign macromolecular substances and nutrients, intracellular endocytosis is a highly conserved process that is instrumental for fungal growth and pathogenic processes^50^, as demonstrated previously in *M. oryzae*^51^ and *U. maydis*^52^. In this study, it was predicted that genes within Contig00011.26 may play an important role in the process of endocytosis. Therefore, the study of this pathway will further our understanding of pathogenic mechanisms of *Sc*.

Protease is considered to be an important virulence factor of pathogenic fungi involving in the process of adhesion, colonization, dissemination and escape of the host during the immune response. In this study, two up-regulated DEGs related to proteases were predicted, namely Contig00012.91 and Contig00022.5. Contig00012.91 was coded Alkaline protease 1 in Swiss_Prot_database with multiple GO function terms, including serine-type endopeptidase activity (GO:0004252), proteolysis (GO:0006508) and identical protein binding (GO:0042802).Contig00022.5 was coded Alpha-lytic protease prodomain with in the process of negative regulation of catalytic activity (GO:0043086), serine-type endopeptidase activity (GO:0004252), extracellular region (GO:0005576) and proteolysis (GO:0006508).

## Conclusions

GSB, a worldwide disease, is easy to infect melon crops. In recent years, GSB has seriously affected pumpkin production in northeast China, where the seed used pumpkin was one of the most important industrial crops, causing huge losses to agricultural producers. Although appropriate cultivation measures can alleviate the disease to some extent, they cannot cure the disease accurately. This is the first report to carry out high-throughput whole-genome sequencing and genome assembly of *Stagonosporopsis cucurbitacearum* (*Sc.*), which was the main pathogenic fungus of pumpkin seedling GSB in northeast China, revealing the pathogenic molecular regulatory mechanism involved in the growth and development of this fungus. Meanwhile, transcriptional analysis of *Sc.* before and after infecting with host was conducted, and differentially expressed disease-related genes and metabolic pathway were explored. This study laid a foundation for the further development of more effective application of pathogenic genes in the development of green prevention and control of new fungicides.

## Materials and methods

### Sampling and isolation of fungi

*Sc.* strain zq-1, obtained from a pumpkin field in northeast China in August 2016, was isolated by Zhao and colleagues as previously described^53^. Briefly, a white colony with limited mycelial expansion was cultured to produce a large colony with irregular edges containing numerous mycelial groupings with mycelial diameters ranging from 15 ~ 20 µm × 5 ~ 6.3 µm, as consistent with known colony morphology of *Sc.* Further confirmation of the identity of the fungus as *Stagonosporopsis cucurbitacearum* was obtained using ITS sequence comparison analysis^53^. PDA solid culture medium was used for strain cultivation. After incubation for 72 h at 28 °C, thalli were scraped from surfaces of agar surfaces and placed in EP tubes without medium and frozen at − 80 °C. Approximately 1 g of *Sc.* thalli (maintained in the frozen state using liquid nitrogen) was ground into a powder that was then subjected to total DNA extraction using a modified CTAB method, as previously described^54^. The ultimate yield of thallus nucleic acid was approximately 7 μg.

### Genome sequencing and assembly

In order to investigate *Sc.* infection mechanisms, genomic DNA was extracted from mycelia and subjected to genome-wide sequencing, assembly and analysis to create an *Sc.* genomic sequence toward meeting the goal of understanding *Sc.* pathogenic mechanisms at the genome level. The *Sc.* genome was sequenced using the Single Molecule Real-Time (SMRT) Kit obtained from Biomarker Technologies (Beijing, China)^55^. DNA libraries with 270-bp or 10-kb inserts were constructed. The 270-bp library was constructed following Illumina’s standard protocol, including fragmentation of genomic DNA, end repair, adaptor ligation and PCR amplification. The 270-bp library was quantified using 2100 Bioanalyzer (Agilent, USA) and subjected to paired-ended 150-bp sequencing using Illumina HiSeq 4000. Sequencing data (filtered reads: 3.21G, sequencing depth: 91×) were used to estimate genome size, repeat content and heterozygosity. Next, the 10-kb library was constructed following PacBio’s standard methods, including fragmentation of genomic DNA, end repair, adaptor ligation and template purification. The 10-kb library was quantified using a 2100 Bioanalyzer (Agilent, USA) and sequenced using SMRT followed by filtering of sequence data (filtered reads: 5.24G, sequencing depth: 148×). The resulting set of high-quality sequences was assembled using Canu v.1.2 using default parameter settings^56^. Finally, Illumina reads were subjected to error correction and gap filling using SOAPdenovo2 with GAPCLOSER v1.12^57^.

### Genome annotation

Protein-encoding genes were annotated using a combination of three independent ab initio gene-finding tools: Augustus, GlimmerHMM and SNAP. Next, GeMoMa^58^ was used to make gene predictions based on gene sequences of analogous species (*Ascochyta rabiei* and *Lm.*). Finally, EVidenceModeler (EVM)^59^ was used to integrate the aforementioned results to obtain final gene prediction results. Next, transcriptome assemblies were mapped to the genome using Trinity then assemblies were subjected to functional annotation to predict protein sequences using GO (https://www.geneontology.org/), Nr (ftp://ftp.ncbi.nih.gov/BLAST/db/), Swiss-Prot (https://www.uniprot.org/), KEGG (www.kegg.jp/kegg/kegg1.html) and COG (https://www.ncbi.nlm.nih.gov/COG/) analyses of BLASTP results with e-values ≤ 1e−5. KOBAS 2.0 software was used for KEGG annotation^60^. HMMER software was used for single gene sequence prediction and to conduct Pfam database comparisons. Domain-calling analyses of protein-encoding genes were performed using the Pfam database^61^ and HMMER. Three functional databases, the Carbohydrate-Active EnZymes database (CAZyme), Transporter Classification Database (TCDB) and Pathogen-Host Interactions database (PHI), were used to deduce functions of proteins predicted from gene sequences using BLAST and to detect potential virulence-related proteins. Genes involved in secondary metabolism, including PKSs (polyketide synthases), NRPSs (non-ribosomal peptide synthetases) and hybrids of these genes (NRPS-PKSs) were identified through whole-genome analysis using SMuRF^62^. Various PKS or NRPS proteins were analyzed using the SBSPKS database server tool to modulate and extract conserved domains via BLAST search^63^.

### Genome comparison

Gene prediction and annotation results for the *Sc.* zq-1 genome were used to determine the distribution of identified fungal genes in selected eukaryotic microbial species. Next, matching genes were counted and searched against the Nr database to generate a distribution map of sequences among genomes of analyzed species. According to the gene distribution map, a large number of *Sc.* genes aligning to *Lm.* sequences were observed, prompting comparison of the whole genomes of both organisms using BLAST. In addition, MCScanX^64^ software was used to conduct collinearity analysis of amino acid sequences of both genomes (using default program parameters) for prediction of genome sequence differences and structural changes that have occurred during evolutionary processes to further clarify the degree of genomic similarity between these two eukaryotic microorganisms.

### Transcriptome analysis

DESeq was used to obtain two differentially expressed gene sets, *Sc.*1 (*Sc.* zq-1 grown on PDA medium) and *Sc.*2 (*Sc.* zq-1 grown on pumpkin leaves). *Sc.1* was stored in a refrigerator at – 20 °C with PE tube slant culture medium. After activation at room temperature, mycelia were transferred to a new plate (20 mL PDA medium) with inoculation ring and continued to grow in a 28 °C light incubator. While *Sc.2* inoculated plant host material "Yinhui 2", is a typical seed pumpkin variety. All samples were immediately frozen and stored in liquid nitrogen until RNA extraction. Fungal samples grown on either PDA or leaves were collected in triplicate to generate a total of six cDNA libraries. To make each library, 3 μg RNA was used to make cDNA using an NEBNextUltraRNA Library Prep Kit for Illumina (NEB, MA, USA) following the manufacturer’s recommendations. Index codes were added to link sequences to their corresponding starting samples. Clustering of index-coded samples was performed using a cBot Cluster Generation System employing a TruSeq PE Cluster Kit v3-cBot-HS (Illumina) according to the manufacturer’s instructions. After cluster generation, library preparations were sequenced on an Illumina Hiseq 2000 platform and paired-end reads were generated. Raw data (raw reads) in FASTQ format were first processed using in-house Perl scripts and Q20, Q30, GC content, and the level of sequence duplication within the clean data were calculated. R package (DESeq 1.10.1, https://www.bioconductor.org/packages/release/bioc/html/DESeq.html) was used for differential expression analysis using the threshold of P < 0.05^65^.

### Analysis of the genome characteristic

Due to the relatively low conservation of repeat sequences among species compared here, a pipeline combining four software tools, LTR FINDER^66^, MITE-Hunter^67^, RepeatScout^68^ and PILER-DF^69^, was used to construct a repeat sequence database of genomic sequence data based on principles of structure prediction and ab initio prediction. The database was classified using PASTEClassifier^70^ then was merged with the Repbase database^71^ to construct the final repeat sequence database. Next, RepeatMasker^72^ software was used to predict repeat sequences from sequencing data. Non-coding RNAs, including various RNAs with known functions (such as rRNA and tRNA), were predicted based on structural characteristics, with different strategies adopted to predict different types of non-coding RNAs, including genome-wide comparisons based on BLASTn search results^73^, identification of rRNA based on matches to Rfam database sequences^74^ and identification of t-RNA sequences using tRNAscan-SE^75^ software. Meanwhile, pseudogenes (non-functional genes) were predicted using gene sequence comparison tool GeneWise^76^ to detect immature termination codons and code shift mutations within gene sequences. SignalP v4.0^77^ and TMHMM^78^ were used to analyze the protein sequences of all the predicted genes and identify the signal peptide proteins and transmembrane proteins, respectively. And secreted proteins were predicted by remove the transmembrane proteins from signal peptide proteins.

### Functional study of predicted pathogenic genes

All products generated via polymerase chain reaction (PCR) amplification using high-fidelity enzyme were digested using suitable restriction enzymes then were ligated to corresponding restriction enzyme-digested vector cloning sites. Recombinant plasmids were then transformed into *Escherichia coli* for plasmid micropropagation. Bacterial colonies containing plasmids with suitable inserts were verified via PCR then were isolated and transformed into *Agrobacterium tumefaciens*. The apoptosis inducer BAX (which can induce a hypersensitive necrosis reaction in tobacco plants) and a characteristic pathogen-associated molecular pattern (PAMP) protein of *Phytophthora* oocytes (infestin-1 or INF1) served as positive controls and GFP as negative control. An agrobacterium-mediated viral expression system was used to instantaneously express effector factors in tobacco plants^30^. Effector factors directly causing plant responses were screened out to allow for detection of candidate fungal effector functions.

### qRT-PCR validation

Differentially expressed genes related to fungal pathogenesis were randomly selected for qRT-PCR analysis. For RNA preparation, *Sc.* samples were collected 5 days post-inoculation from fungal cultures growing on PDA medium or on pumpkin leaves then total RNA was prepared using a PrimeScript RT reagent Kit with gDNA Eraser (Perfect Real Time) (Takara, Otsu, Japan). 1 μg of total RNA as template was reverse transcribed to generate cDNA using instructions provided with the kit. Next, qRT-PCR was performed for biological samples in triplicate using an ABI PRISM 7500 fast real-time PCR system (Applied Biosystems, Foster City, CA, USA) and thermal cycling conditions as follows: 1 cycle of initial denaturation at 95 °C for 5 min followed by 35 cycles of: denaturation at 95 °C for 15 s, annealing at 60 °C for 15 s and extension at 72 °C for 15 s. Finally, the t-test was used to conduct difference analysis of data to assess if expression level differences were statistically significant based on the threshold p < 0.05. Primers used in qRT-PCR for DEGs validation are shown in Supplementary Table S13 .

### Ethical approval

The authors note that this research was performed and reported in accordance with ethical standards of scientific conduct.

## Supplementary information

Supplementary Figure 1.

Supplementary Figure 2.

Supplementary Figure 3.

Supplementary Figure 4.

Supplementary Figure 5.

Supplementary Figure 6.

Supplementary Figure 7.

Supplementary Figure 8.

Supplementary Legends.

Supplementary Tables.
