# Supplementary figures and images for: Genome-scale analyses and characteristics of putative pathogenicity genes of Stagonosporopsis cucurbitacearum, a pumpkin gummy stem blight fungus
Source: Sci Rep. 2020 Oct 22;10:18065. doi: 10.1038/s41598-020-75235-x (PMC7581720; doi:10.1038/s41598-020-75235-x)

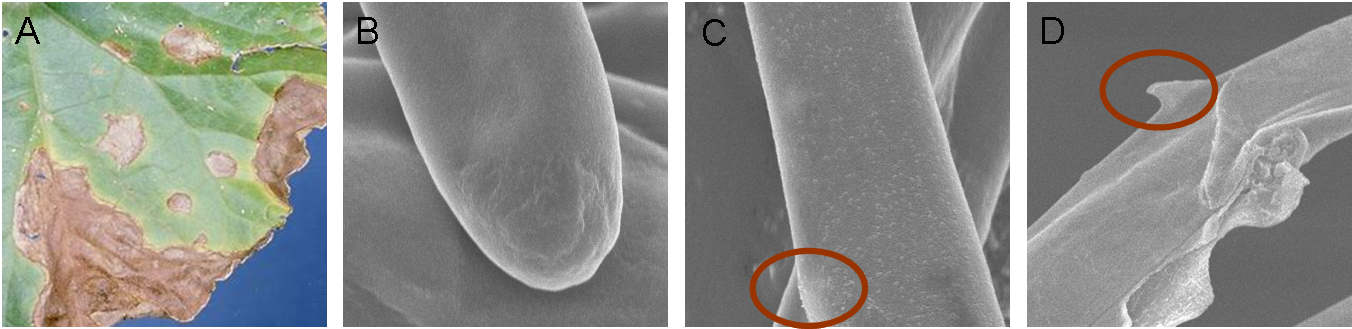

Supplement: Supplementary file 1 — Supplementary Figure 1. [file 41598_2020_75235_MOESM1_ESM.tiff]

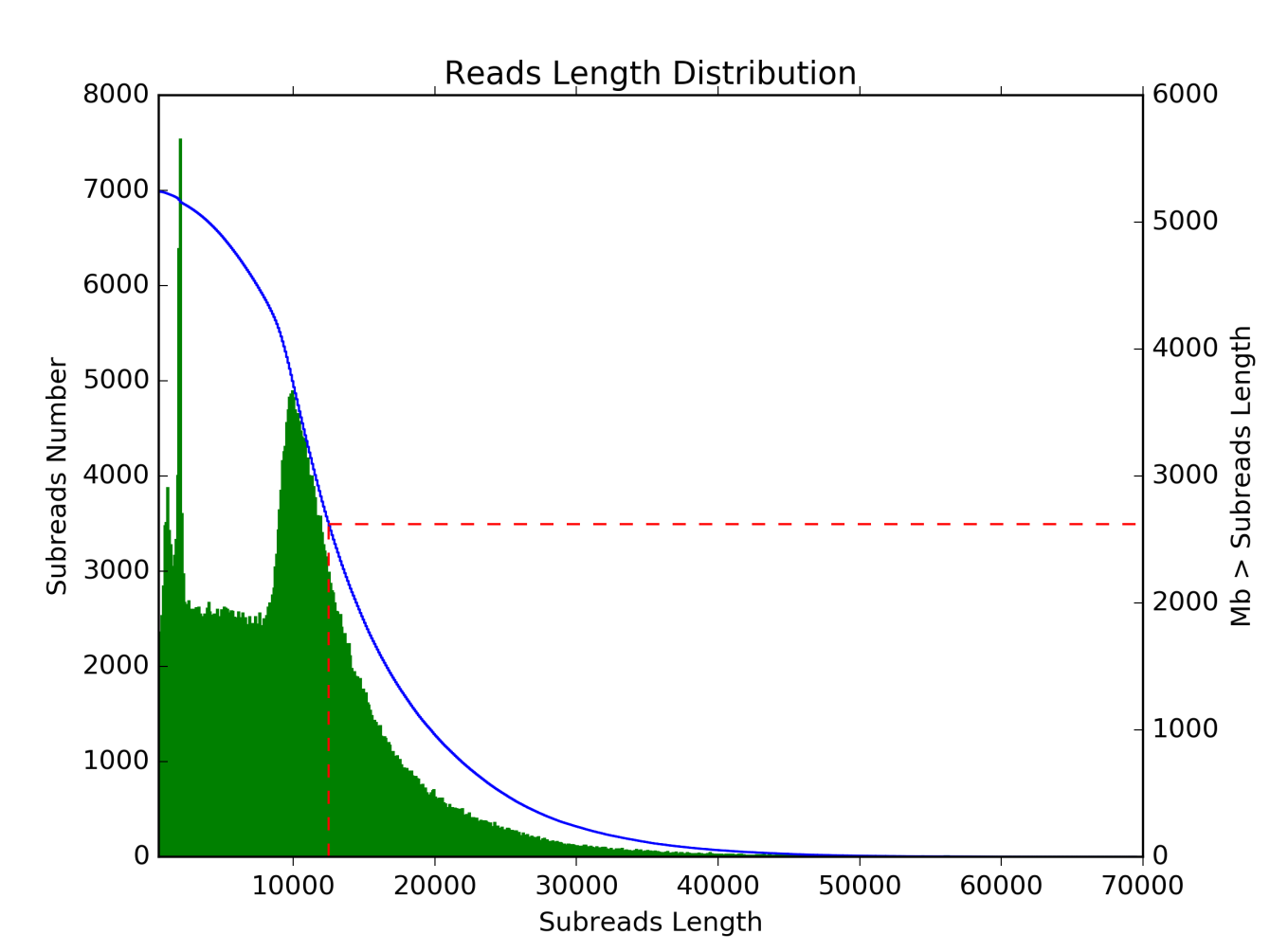

Supplement: Supplementary file 2 — Supplementary Figure 2. [file 41598_2020_75235_MOESM2_ESM.tiff]

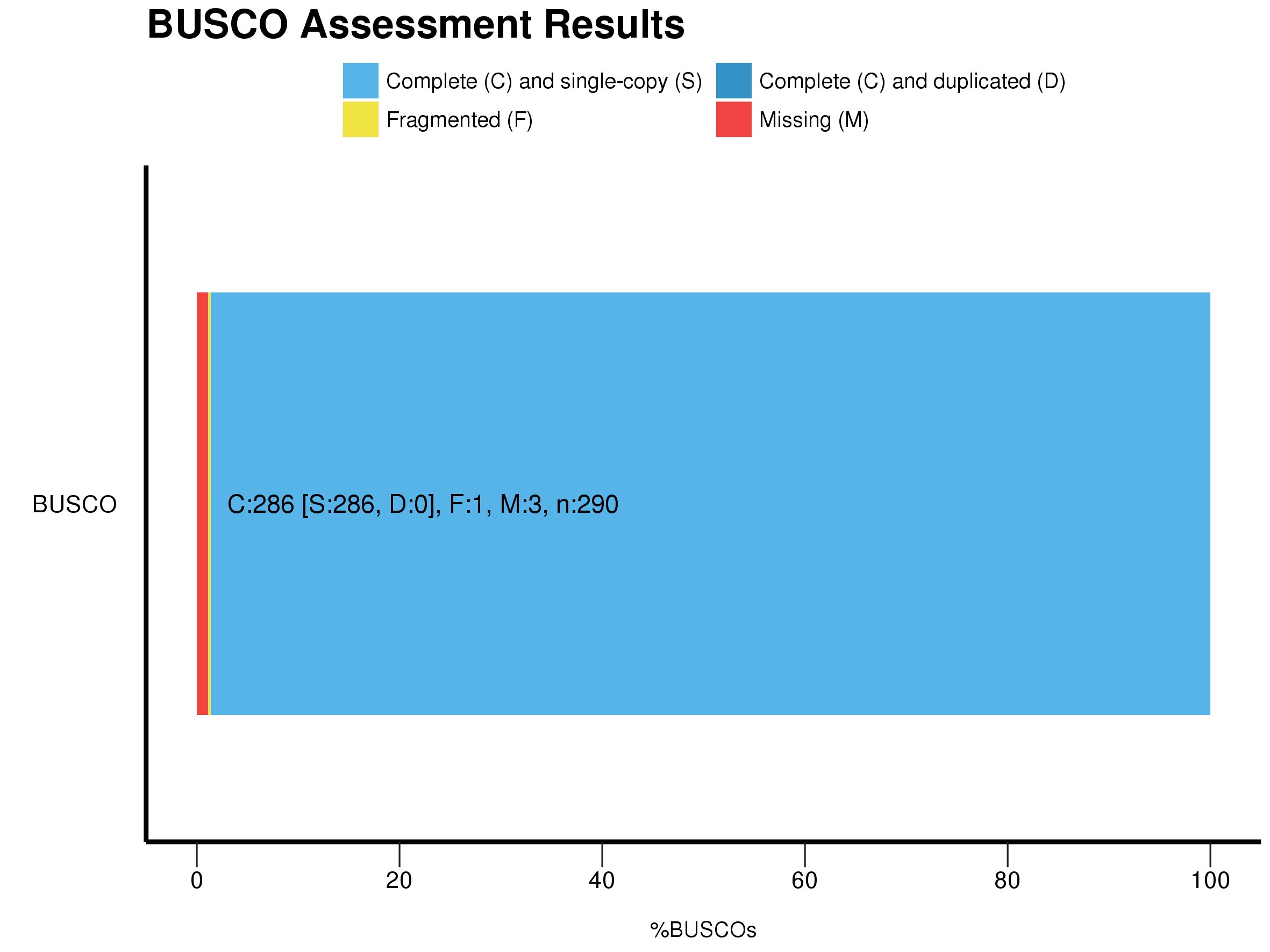

Supplement: Supplementary file 3 — Supplementary Figure 3. [file 41598_2020_75235_MOESM3_ESM.tiff]

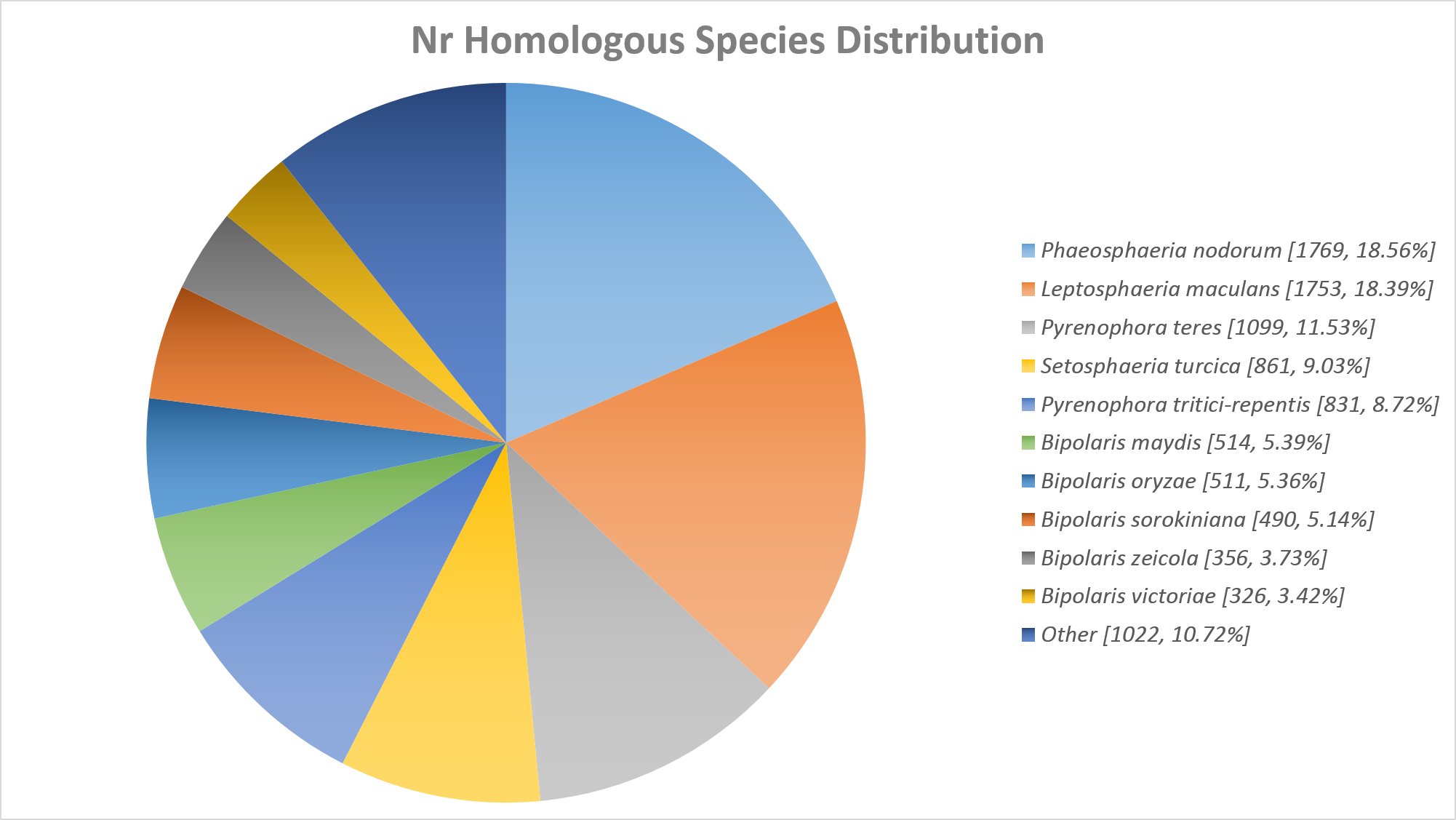

Supplement: Supplementary file 4 — Supplementary Figure 4. [file 41598_2020_75235_MOESM4_ESM.tiff]

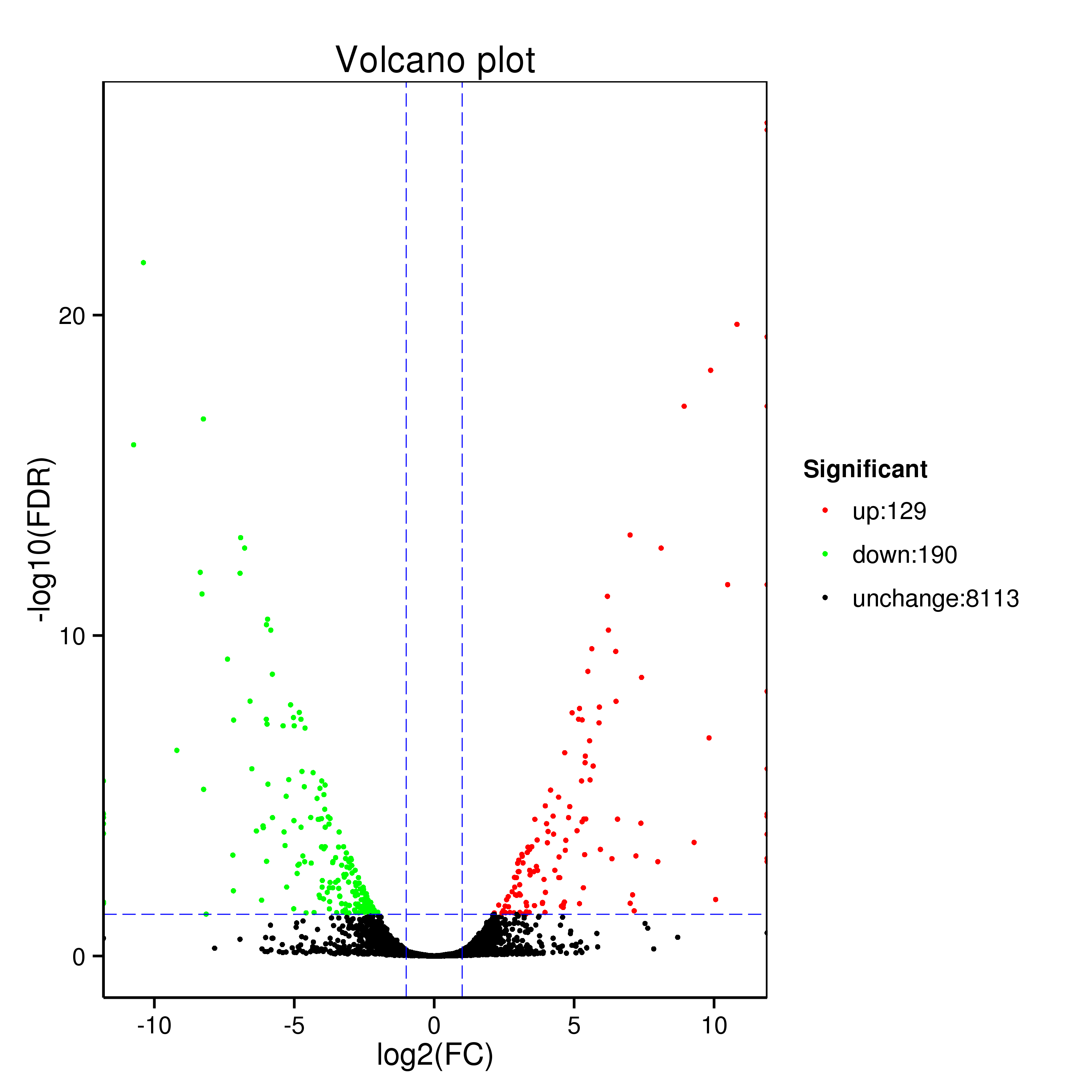

Supplement: Supplementary file 5 — Supplementary Figure 5. [file 41598_2020_75235_MOESM5_ESM.tiff]

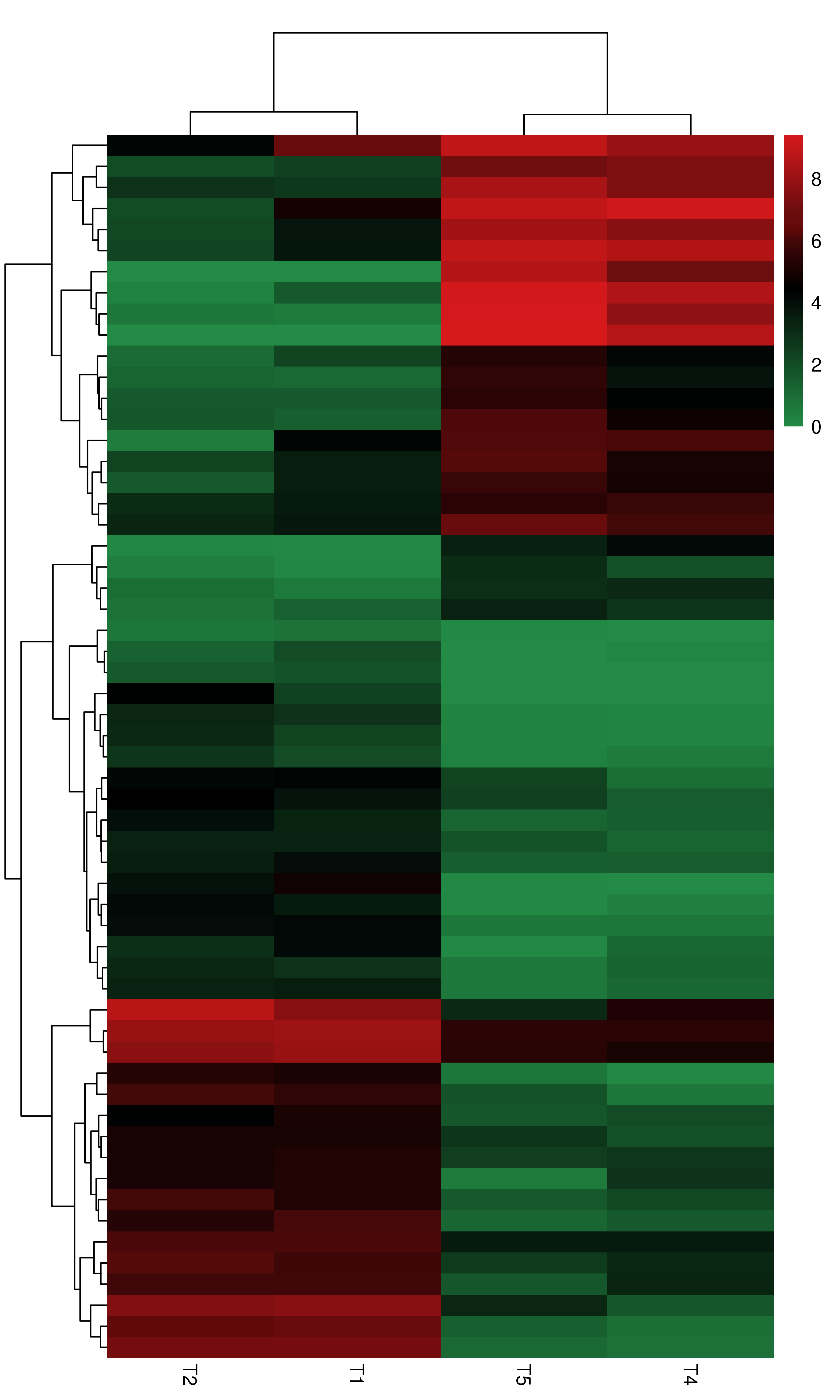

Supplement: Supplementary file 6 — Supplementary Figure 6. [file 41598_2020_75235_MOESM6_ESM.tiff]

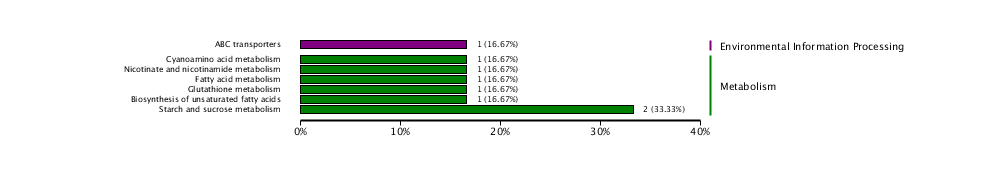

Supplement: Supplementary file 7 — Supplementary Figure 7. [file 41598_2020_75235_MOESM7_ESM.tiff]

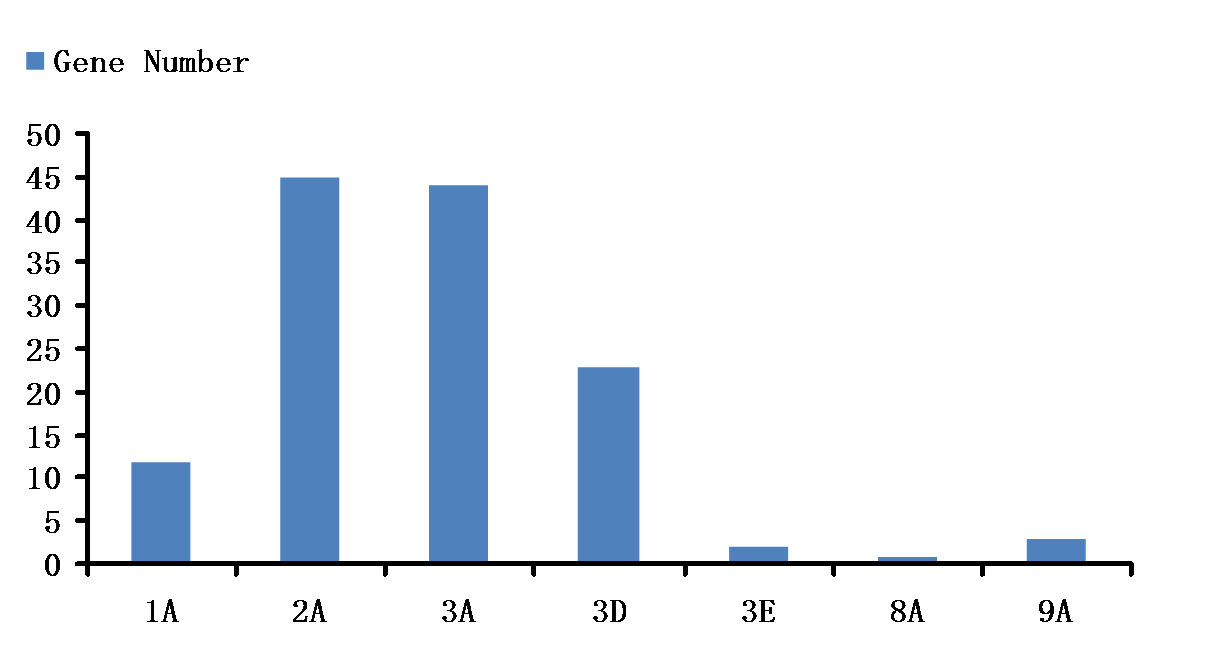

Supplement: Supplementary file 8 — Supplementary Figure 8. [file 41598_2020_75235_MOESM8_ESM.tiff]
