# Supplementary material for: Genome-scale analyses and characteristics of putative pathogenicity genes of Stagonosporopsis cucurbitacearum, a pumpkin gummy stem blight fungus
Source: Sci Rep. 2020 Oct 22;10:18065. doi: 10.1038/s41598-020-75235-x (PMC7581720; doi:10.1038/s41598-020-75235-x)
Supplement: Supplementary file 9 — Supplementary Legends. [file 41598_2020_75235_MOESM9_ESM.docx]

# Supporting Information captions

***S1* Table. Statistical results of the filtered *Sc*. genome sequencing data.**

***S2* Table. Statistics of gene functional annotation.** ***S3* Table. Informations of RNA-seq data for *Sc.1* and *Sc.2*. "*Sc. 1*" （T1, T2, T3）and "*Sc. 2*"（T4, T5, T6） representing the samples of before and after infection, respectively.*S4* Table. 58 DEGs were identified to relative to the** [**Pathogenicity**](javascript:;) **by GO analysis.**

***S5* Table. Statistics of functional domains of major secreted proteins in the *Sc*. genome. *S6* Table. 54 common elements in "SP genes" and "DEGs".**

***S7* Table.** **96 common elements in "PHI genes" and "DEGs".**

***S8* Table. 36 common elements in "Carb-coded genes" and "DEGs".**

***S9* Table. Database analysis annotation statistics of GH109 family in Sc. genome.**

***S10* Table. Database analysis annotation statistics of GH18 family** **in *Sc.* genome.**

***S11* Table. Annotation of putative key enzymes genes of secondary metabolisms.**

***S12* Table. Annotation of putative NRPS, NRPS-Like and T1PKS by functional domains.**

***S13* Table. TCDB and PHI database joint analysis annotated statistics.**

***S14* Table*.* The primers used in qRT-PCR for putative pathogenic DEG validation.*S1* Fig*.* Disease symptoms of *Sc.* leaf and observation of infection nails.**

(A) Disease symptoms of *Sc.* leaf.

(B) The characteristics of the uninfected host.

(C) The hyphae characteristics were infected host for 12 hours.

(D) The hyphae characteristics were infected host for36 hours.

***S2* Fig. Statistics analysis for high quality reads (subreads) of *Sc.* genome sequencing data.**

Total base length is 5,242,297,168 with estimated genome coverage ≥100× were obtained. The mean subreads length is 9946 bp and subreads number is 527,073.

***S3* Fig. BUSCO Assessment Results.**

***S4* Fig. *Sc.* and analogous *S*pecies distribution map of Nr database alignment to sequence.** Reflecting the distribution of analogous species in Nr annotated genome, different colors represent different analogous species. Percentage is the proportion of similar sequence statistics of a species in the total number of sequences of that species.

***S5* Fig. Volcano plot of DEGs.** Each point of the volcano plot of DEGs represents one gene; the X-coordinate indicates the log value of the fold-change in expression level of a given gene between the two samples, with a larger absolute value corresponding to a larger fold-change of expression quantity between samples. The Y-coordinate indicates the negative log value of the error detection rate, with a larger value corresponding to greater significance of differential expression, and therefore greater reliability of screened DEGs. The green and red points in the diagram represent significantly differentially expressed genes: green points represent down-regulated genes; red points represent up-regulated genes; black points represent no significance between gene expression levels.

***S6* Fig. Clustergram of expression patterns of 58 DEGs were identified to relative to the** [**pathogenicity**](javascript:;) **by GO analysis.** Here expression levels of 58 DEGs are shown, including T01, T02, T04 and T05. The lines shown in the diagrams represent different samples and different rows represent different genes. Color coding indicates the log value of gene expression quantity FPKM using a base value of 2.0. TBtools was used for drawing program.

***S7* Fig. The KEGG classification map of 10 pathogenic DEGs.** The Y-coordinate represents the KEGG metabolic pathway and the X-coordinate represents the number of genes within each pathway and also the percentage of annotated genes of each pathway per total number of genes.

***S8*** **Fig. The number of genes in each transport protein secondary classification*.*** In the abscissa, 1A, 2A, 3A, 3D, 3E, 8A and 9A represent the biochemical mechanisms of channels, transporters (unidirectional transporters, co-transporters, and reverse transporters), p-p phosphorylation-driven transporters, redox-driven transporters, phosphorylation-driven transporters, auxiliary transporters, and unknown transporters, respectively.
